# Switch of innate to adaptative immune responses in the brain of patients with Alzheimer’s disease correlates with tauopathy progression

**DOI:** 10.1038/s41514-024-00145-5

**Published:** 2024-03-18

**Authors:** Marcos R. Costa

**Affiliations:** 1 Univ. Lille, Inserm, CHU Lille, Institut Pasteur de Lille, U1167-RID-AGE facteurs de risqué et déterminants moléculaires des maladies liées au vieillissement, DISTALZ, 1 rue du Professeur Calmette, 59019 Lille, France; 2 Brain Institute, Federal University of Rio Grande do Norte, Natal, Brazil

**Keywords:** Alzheimer's disease, Alzheimer's disease

## Abstract

Neuroinflammation is a key feature of Alzheimer’s disease (AD). In this work, analysis of single- cell RNA-sequencing (scRNA-seq) data obtained from the brain of patients with AD provides evidence supporting a switch from an innate to an adaptative immune response during tauopathy progression, with both disease-associated microglia (DAM) and CD8+ T cells becoming more frequent at advanced Braak stages.

## Introduction

In the brain of patients with Alzheimer’s disease (AD), both extracellular deposition of amyloid-β as neuritic plaques and intracellular accumulation of hyperphosphorylated tau as neurofibrillary tangles are associated with innate and adaptative immune responses^1–4^. Similarly, inflammatory responses are observed in virtually every animal model of amyloidopathy and tauopathy^5^, indicating an intimate interaction between these pathological processes. However, the influence of different immune cell types/subtypes to AD pathology onset and progression remains largely unknown.

Animal models of tauopathy (Tau(P301S)) develop a unique innate and adaptive immune response in the brain, characterized by a marked increase in the numbers of T cells, especially cytotoxic T lymphocytes, and correlated with the extent of neuronal loss^6^. Depletion of T cells in Tau(P301S) mice via intraperitoneal administration of anti-CD4 and anti-CD8 antibodies is sufficient to ameliorate brain atrophy and improves behavior, suggesting that infiltration of T cells in the brain parenchyma is a key step in neurodegeneration. Infiltration of CD8+ T cells into stem cell-derived neurons, astrocytes and microglia cultures also leads to increased neurodegeneration^7^. However, it remains unclear whether T cell infiltration correlates with AD pathology progression in the human brain.

In this work, single-nucleus RNA-sequencing (snRNA-seq) data obtained from the brain of patients with AD and age-matched controls^8^ was exploited aiming to characterize the innate and adaptative immune responses at different pathological stages.

## Results

### Switch of innate to adaptative immune responses in the brain of patients with AD

**Fig. 1:**
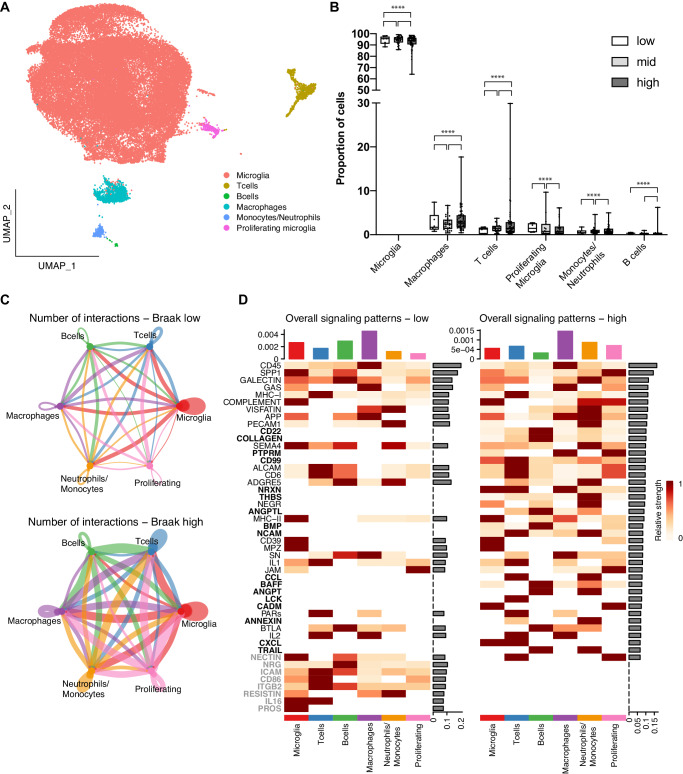
Increased proportion of adaptative immune cells in the brain of patients with late-stage AD. **A** UMAP representation of the different immune cell types in the brain of AD and age matched control subjects identified using snRNA-seq. **B** Box plot showing the proportion of the different cell types in subject’s brains pathologically classified at low, mid and high Braak stages (****p < 0.0001; GLM). Whiskers indicate minimum and maximum, dots indicate the percentage of cells in each subject and asterisks indicate statistical significance for 1, 2 or 3 pair of comparisons. **C** Circle plots showing the number of intercellular interactions among immune cells at early- (Braak low) and late-stages (Braak high) of tauopathy. Edge colors are consistent with the sources as sender, and edge weights are proportional to the interaction strength. Circle sizes are proportional to the number of cells in each cell group. **D** Heatmap showing the overall signaling patterns identified. Pathways highlighted in bold are upregulated, whereas those highlighted in gray are downregulated at late stages of tauopathy (Braak high). The top-colored bar plot represents the communication probability, and the right bar plot represents the sum of row of values (relative strength).

To investigate the cellular dynamics associated with innate and adaptative immune responses in the brain of patients with different degrees of tauopathy, microglia and white blood cell populations from the middle temporal gyrus (MTG) of 42 elderly individuals diagnosed with dementia and 42 age-matched healthy individuals^8^ were analyzed. Unsupervised clustering identified 26 immune cell clusters expressing PTPRC (CD45) that could be annotated using a combination of cell markers such as ITGAM (CD11B), TREM2, P2RY12 (microglia), CD3D, CD3G (T cells), CD19, JCHAIN (B cells), MRC1 (macrophages), S100A8, FCGR3A, NR4A1 (monocytes/neutrophils), and other genes differentially expressed in each cluster compared to all others (Fig. 1, Supplementary Fig. 1, Supplementary Table 1). Based on these profiles, clusters of microglial cells, T cells, B cells, macrophages, monocytes and neutrophils were identified (Fig. 1A; Supplementary Fig. 1). Comparison of the proportion of immune cells in patients’ brains pathologically classified at different Braak stages (0 to II – “low”; III to IV – “mid”; V to VI - “high”) using a generalized linear model (GLM) revealed that the proportion of microglial cells was significantly lower in mid vs low, as well as in high vs mid and high vs low Braak stage comparisons (Fig. 1B; Supplementary Table 2). Conversely, the proportions of all adaptive immune cells were significantly higher in mid vs low and high vs mid Braak stage comparisons (Fig. 1B), indicating a progressive shift from innate to adaptative immune responses during tauopathy progression in the brain of AD patients.

### Increased intercellular communication among immune cell populations in AD

To identify possible alterations in signaling pathways that could help to explain the shift in the composition of immune cell populations in the brain of AD patients, intercellular communication networks were quantitatively inferred from snRNA-seq data using CellChat^9^. The number of intercellular communication pathways among immune cells increased from 192 in brains with low tauopathy to 486 in those with high tauopathy (Fig. 1C). These interactions could be classified into 48 signaling patterns, of which 17 were up regulated and 8 were down regulated in high compared to low Braak stages (Fig. 1D), including the chemokine (C-X-C motif) ligand (CXCL) and the chemokine (C-C motif) ligand (CCL) pathways (Fig. 1D).

### Progressive increase in the proportion of T cell populations in the brain of patients with AD

**Fig. 2:**
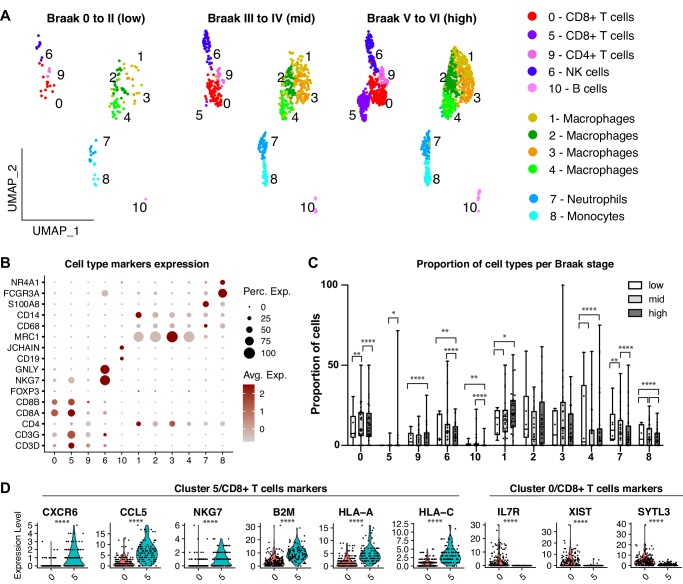
Identification of activated CD8+ T cells in the brain of AD patients at late stages of tauopathy. **A** UMAP representations of the different adaptative immune cell types and subtypes in the brains of subjects at different Braak stages. **B** Dot plot showing the expression of cell-specific markers. **C** Box plot showing the proportion of the different cell types in subject’s brains pathologically classified at low, mid and high Braak stages (*p < 0.05; **p < 0.01; ***p < 0.001; ****p < 0.0001; GLM). Whiskers indicate minimum and maximum, dots indicate the percentage of cells in each subject and asterisks indicate statistical significance for 1, 2 or 3 pair of comparisons. **D** Violin plots showing the expression of genes differentially expressed in CD8+ T cell subtypes (****p < 0.0001; Wilcoxon test).

To further characterize the adaptative immune response in the human brain with different degrees of tauopathy, cell types associated with this response were separated from the complete dataset and reanalyzed using unsupervised clustering (Fig. 2A). Eleven cell clusters could be identified and annotated into 8 major cell types/subtypes based on the expression of top markers (Supplementary Table 3) and cell-type specific markers such as CD3D, CD3G, CD8A and CD8B (CD4+ and CD8+ T cells), NKG7 and GNLY (natural killer – NK cells), CD19, JCHAIN (B cells), MRC1, CD68 and CD14 (macrophages), S100A8, CD68 (neutrophils), FCGR3A and NR4A1 (monocytes) (Fig. 2A, B). Cells from cluster 0 (CD8+ T cells) were 1.56 times more common at mid compared to low Braak stages whereas cells from cluster9 (CD4+ T cells) and 10 (B cells) became progressively more common in brains at mid and high compared to lower Braak stages (Fig. 2C, Supplementary Table 4). The cluster 5 (CD8+ T cells) was not found in brains at low Braak stages but could be detected in one brain at mid Braak stage and 5 brains at late Braak stages (Fig. 2C). Among the genes differentially expressed in CD8+ T cells from cluster 5 compared to cluster 0, a strong up-regulation of activated (CXCR6, CCL5, NKG7, HLA-A and HLA-C) and a downregulation of naïve CD8+ T cells markers (IL7R, XIST and SYTL3) could be observed (Fig. 2D; Supplementary Table 5). Together, these findings suggest that T cells shift from naïve to activated states in the human brain with Tau pathology.

### Shift of T cell states correlates with the presence of DAM populations in the brain of patients with AD

**Fig. 3:**
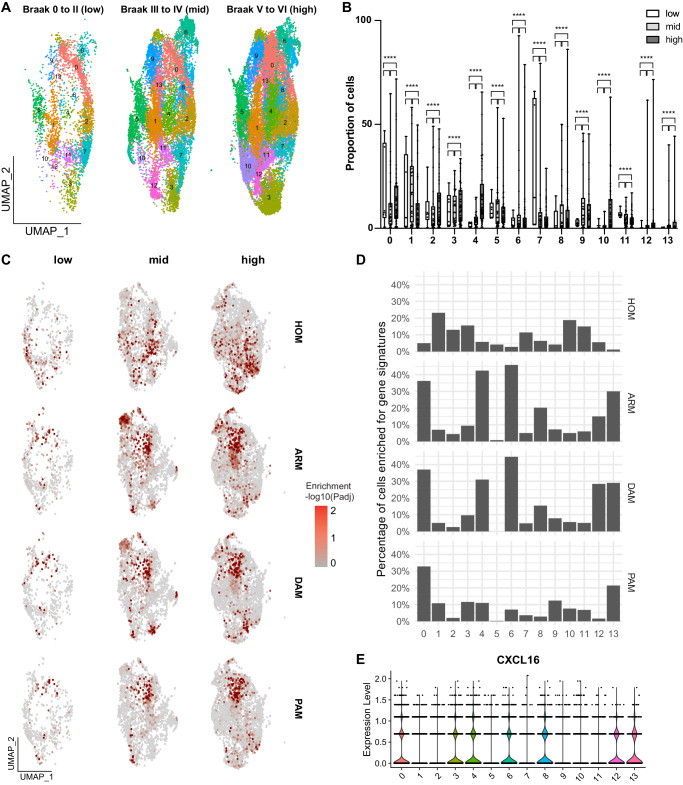
Dynamics of microglial states in the brain of AD patients at different stages of tauopathy. **A** UMAP representations of the different microglial subtypes/states in the brains of subjects at different Braak stages. **B** Box plot showing the proportion of the different cell types in subject’s brains pathologically classified at low, mid and high Braak stages (****p < 0.0001; GLM). Whiskers indicate minimum and maximum, dots indicate the percentage of cells in each subject and asterisks indicate statistical significance for 1, 2 or 3 pair of comparisons. **C** Feature plots showing the enrichment for HOM, ARM, DAM and PAM gene signatures in microglial cells. **D** Proportion of cells significantly enriched for those signatures per cluster. **E** Violin plot showing the predominant expression of CXCL16 in clusters enriched for DAM signatures.

To characterize microglial states associated with Tau pathology in the brain of patients with AD, unsupervised clustering of microglial cells from the original dataset was performed, allowing the identification of 14 microglial clusters (Fig. 3A). The relative proportion of cells in all these clusters significantly changed according to Braak stages (Fig. 3B), indicating a high dynamic of microglial states during Tau pathology progression. The odds ratio of cells belonging to clusters 2, 4, 5, 6, 9, 10, 12 and 13 showed the higher increments (up to 2- to 38-folds) between Braak stages (Supplementary Table 6).

Next, CellID^10^ was employed to extract microglia gene signatures at single-cell resolution using a large set of genes previously identified in well-defined conditions^2,4,11^. This strategy helps to circumvent potential limitations in microglial subset classification/annotation based exclusively on a small set of markers. CellID was employed to calculate the enrichment for gene signatures previously associated with homeostatic (HOM; *n* = 484 genes), disease-associated microglia (DAM; *n* = 306 genes^2^), plaque-associated microglia (PAM; *n* = 57 genes^4^) and activated responsive microglia (ARM; *n* = 131 genes^11^) (Supplementary Table 7) in each microglial cell (Fig. 3C). About one third of cells within clusters 0, 4, 6 and 13 were significantly enriched for ARM and DAM signatures (Fig. 3D). Clusters 0 and 13 also showed more than 20% of cells enriched for PAM signatures at the same time, whereas cluster 12 showed about 30% of cells enriched for DAM signature, 15% of cells enriched for ARM and less than 1% of cells enriched for PAM (Fig. 3D). Thus, among the 8 microglial states showing the greatest increments in proportion according to Braak stages, five harbored ARM-, DAM- and PAM-signature enriched subtypes. Interestingly, all clusters harboring cells enriched for those signatures expressed high levels of CXCL16 (Fig. 3E), suggesting that activated microglia could mediate T cell trafficking into the brain via the CXCL16-CXCR6 signaling axis.

## Discussion

Neuroinflammation is a key pathological hallmark of AD^12,13^. Several lines of evidence suggest that inflammatory response in AD has a dual function, playing a neuroprotective role during early stages of disease, but becoming detrimental at later stages when a chronic response is mounted^14^. The present work provides evidence suggesting that the inflammatory response in the brain of patients with AD switches from an innate to an adaptative immune response during disease progression (indicated by postmortem Braak staging). This switch is mainly characterized by an increase in DAM and CD8+ T cells, and could suggest that these cell populations contribute to the detrimental effects of neuroinflammation, as previously suggested in animal models of AD^2,6,15^.

The data presented here also support the role of CXCL16-CXCR6 pathway in the trafficking of CD8+ T cell to the brain parenchyma. CXCL16 is a pleiotropic protein that functions as a chemoattractant for CXCR6-expressing T effector memory cells^16^. Monocytes-derived CXCL16 is elevated in the CSF of cognitively impaired subjects and associate with neuroaxonal damage^17^. The analyses reported here suggest that activated microglial cells may also release CXCL16, thus potentially contributing to CD8+ T cell trafficking to the brain parenchyma. In agreement with this hypothesis, transcriptomic profiles from brain tissues of AD patients and mouse models support an active role of dysregulated CXCL16 during AD pathology progression^18–20^. In the future, it would be interesting to investigate whether the pharmacological blockage of the CXCL16-CXCR6 pathway could reduce the microglia-mediated T cell trafficking in the brain and mitigate the neurodegeneration associated with tauopathy in animal and human neuroimmune axis models^6,7^.

This work also confirms the role of DAM in AD pathology progression. These cells represent transcriptionally distinct microglial profiles observed in several animal models of neurodegeneration and in the diseased human brain^2,4,21^. Distinct Aβ and tau-associated DAM signatures have been described in AD patients^22^ and could explain the different DAM clusters identified in this study. Yet, CXCL16 expression was similar among the different clusters of DAM identified in this study, suggesting that they could equally contribute to T cell infiltration and neurodegeneration.

Altogether, this work provides evidence supporting the view that at least some types of activated microglia observed at early stages of AD pathology could contribute to the infiltration of T effector cells in the human brain parenchyma, potentially contributing to neurodegeneration.

## Methods

### Analysis of single-cell RNA-sequencing data

Data used in this study was obtained from the Seattle Alzheimer’s Disease Brain Cell Atlas (SEA-AD) consortium, which includes the Allen Institute for Brain Science, the University of Washington, and Kaiser Permanente Washington Research Institute. SEA-AD is supported by the National Institutes on Aging (NIA) grant U19AG060909. Study data were generated from postmortem brain tissue obtained from the University of Washington BioRepository and Integrated Neuropathology (BRaIN) laboratory and Precision Neuropathology Core, which is supported by the NIH grants for the UW Alzheimer’s Disease Research Center (P50AG005136 and P30AG066509) and the Adult Changes in Thought Study (U01AG006781 and U19AG066567). The ACT study is a longitudinal population-based prospective cohort study of brain aging and incident dementia in the Seattle metropolitan area. ACT is a repository at the Kaiser Permanente Washington Health Research Institute, which has established policies and procedures for sharing data with external investigators. Data available from this study web site do not require any additional Institutional Review Board (IRB) approval or permissions. Transcriptomic data from 40,000 cells originally annotated as microglia/peri-vascular macrophages (Micro/PVM) was downloaded from the Chan Zuckerberg CELL by GENE - https://cellxgene.cziscience.com/collections/1ca90a2d-2943-483d-b678-b809bf464c30.

Using Seurat, cells from “Reference” were removed from the complete dataset. Then, SCT normalization and unsupervised clustering of the remaining 38,905 cells was performed identifying 16 separate clusters containing at least 50 cells. Cell type/subtype annotation was performed based on the expression of selected markers described throughout the results section. To quantitatively infer and analyze intercellular communication networks from scRNA-seq data, we used CellChatDB^9^. Classification of microglial subtypes/states was performed using CellID^10^. Comparison of cell type/subtype abundance was performed using generalized linear models (GLMs) to make predictions for cell type proportions that depend on predictors of interest (e.g., Braak stage), as well as accounting for batch effects and potential co-variables, such as APOE status, sex and age at death. Other available metadata were also tested as potential co-variables in the GLM but did not change the results. Therefore, they were not included in the final model. Odds ratios were calculated using emmeans and summary statistics for the different comparisons performed in this work are provided as supplementary tables.

### Reporting summary

Further information on research design is available in the Nature Research Reporting Summary linked to this article.

### Supplementary information

Supplementary material

Supplementary Table 1

Supplementary Table 2

Supplementary Table 3

Supplementary Table 4

Supplementary Table 5

Supplementary Table 6

Supplementary Table 7

Reporting Summary

## Data Availability

Transcriptomic data used in this work is available at https://cellxgene.cziscience.com/collections/1ca90a2d-2943-483d-b678-b809bf464c30.

## Code availability

The complete code to reproduce the analyses described in this work is provided as supplementary material.
