# Supplementary material for: Switch of innate to adaptative immune responses in the brain of patients with Alzheimer’s disease correlates with tauopathy progression
Source: NPJ Aging. 2024 Mar 18;10(1):19. doi: 10.1038/s41514-024-00145-5 (PMC10948755; doi:10.1038/s41514-024-00145-5)
Supplement: Supplementary file 1 — Supplementary material [file 41514_2024_145_MOESM1_ESM.pdf]

Switch of innate to adaptative immune responses in the brain of patients with Alzheimer's disease correlates with tauopathy progression

Marcos R. Costa

Supplementary Information

Supplementary methods

R script to reproduce analyses

```
library(dplyr)

library(Seurat)

library (Matrix)

library(patchwork)

library(data.table)

library(readr)

library(tidyverse)

library (ggplot2)


# Download and open the RDS object "Micro-PVM - MTG" at
https://cellxgene.cziscience.com/collections/1ca90a2d-2943-483d-b678-b809bf464c30

SEAAD_Micro <- readRDS("~/Downloads/local.rds")


# SCTransform and remove confounding sources of variation

SEAAD_Micro <- SCTransform (SEAAD_Micro, assay = "RNA")


# Perform linear dimension reduction

SEAAD_Micro <- RunPCA(SEAAD_Micro, features = VariableFeatures(object = SEAAD_Micro))


# Clustering cells

# Repeat downstream analyses with a different number of PCs (10, 15, or even 50!).As you will
observe, the results often do not differ dramatically.

# Resolution between 0.4-1.2 typically returns good results for single-cell datasets of around 3K cells.
```

```
SEAAD_Micro <- RunUMAP(SEAAD_Micro, reduction = "pca", dims = 1:20)

SEAAD_Micro <- FindNeighbors(SEAAD_Micro, dims = 1:20)

SEAAD_Micro <- FindClusters(SEAAD_Micro, resolution = 1)


DimPlot(SEAAD_Micro, reduction = "umap", label=TRUE)


# Number of cells per cluster

cellInfo <- data.frame(seuratCluster=Idents(SEAAD_Micro))

cbind(table(cellInfo$seuratCluster))


# Save the object at this point so that it can easily be loaded back in

saveRDS(SEAAD_Micro, file = "SEAAD_Micro_sct.rds")


# Classify cells based on cluster markers

Idents (SEAAD_Micro)<-SEAAD_Micro@meta.data$SCT_snn_res.1

DimPlot(SEAAD_Micro, reduction = "umap", group.by="SCT_snn_res.1", label=T)

SEAAD_Micro.markers <- FindAllMarkers(SEAAD_Micro, only.pos = TRUE, min.pct = 0.25,
logfc.threshold = 0.25)

SEAAD_Micro.markers %>% group_by(cluster) %>% top_n(n = 2, wt = avg_log2FC)

write.csv(SEAAD_Micro.markers, "Markers_Allimmuno.csv")


# Top 10 genes

top20 <- SEAAD_Micro.markers %>% group_by(cluster) %>% top_n(n = 20, wt = avg_log2FC)

DoHeatmap(SEAAD_Micro, features = top20$gene) + NoLegend()


DotPlot (SEAAD_Micro, features=c("PTPRC","ITGAM", "TREM2","P2RY12", "MKI67","CD3D", "CD3G",
"CD19", "JCHAIN", "MRC1", "S100A8",

                              "FCGR3A",  "NR4A1"), cols= c("lightgrey", "red"), group.by="SCT_snn_res.1",
assay="RNA") + coord_flip()


# Assigning cell type identities
```

```
new.cluster.ids <- c("Microglia", "Microglia", "Microglia", "Microglia", "Microglia", "Microglia",
"Microglia",
                "Microglia", "Microglia", "Microglia", "Microglia", "Microglia", "Microglia", "Microglia",
                "Microglia", "Macrophages", "Microglia", "Microglia", "Tcells", "Microglia", "Microglia",
                "Microglia", "Proliferating", "Microglia", "Monocytes/Neutrophils", "Tcells", "Bcells")
names(new.cluster.ids) <- levels(SEAAD_Micro)
SEAAD_Micro <- RenameIdents(SEAAD_Micro, new.cluster.ids)
DimPlot(SEAAD_Micro, reduction = "umap", label = TRUE, pt.size = 0.5) + NoLegend()

# Create a metadata column for cell types
SEAAD_Micro@meta.data$Main.Groups <- SEAAD_Micro@active.ident

saveRDS(SEAAD_Micro, file = "SEAAD_immuno.rds")

# remove "Reference"
SEAAD_immuno <- subset (SEAAD_immuno, Braak.stage=="Reference", invert=TRUE)

# Create Braak low, mid and high metadata
Idents(SEAAD_immuno)<- SEAAD_immuno@meta.data$Braak.stage
SEAAD_immuno<-RenameIdents(SEAAD_immuno, "Braak 0"="low")
SEAAD_immuno<-RenameIdents(SEAAD_immuno, "Braak II"="low")
SEAAD_immuno<-RenameIdents(SEAAD_immuno, "Braak III"="mid")
SEAAD_immuno<-RenameIdents(SEAAD_immuno, "Braak IV"="mid")
SEAAD_immuno<-RenameIdents(SEAAD_immuno, "Braak V"="high")
SEAAD_immuno<-RenameIdents(SEAAD_immuno, "Braak VI"="high")

SEAAD_immuno$Braak <-SEAAD_immuno@active.ident
SEAAD_immuno$Braak <- factor(SEAAD_immuno$Braak, levels=c("low", "mid", "high"))
Idents(SEAAD_immuno)<- SEAAD_immuno@meta.data$Main.Groups
```

Proportion of cells per disease condition or stage

```
mtd<-data.table(SEAAD_immuno@meta.data,keep.rownames = "bc")

ggplot(mtd)+geom_bar(aes(x=Braak ,fill=Main.Groups),position = "fill")

mtd[,nsample:=.N,by=c("donor_id")]
mtd[,pct.ct:=.N/nsample,by=c("Main.Groups", "donor_id")]
p<-ggplot(unique(mtd[,.(Braak,donor_id,pct.ct,Main.Groups)]),aes(x=Braak,y=pct.ct,col=donor_id))+
  geom_jitter()+
  stat_summary(fun= "mean",colour="red")+
  facet_wrap("Main.Groups",scales = "free_y")+
  scale_y_continuous(labels=scales::percent)+theme_minimal() + NoLegend()
ggsave("SEAAD_immuno_Distrib_jitter_samples_Main.Groups_Braak.pdf")
write.csv (p$data, "SEAAD_immuno_proportion_clusters_Braak.csv")

library("ggpubr")

ggboxplot(mtd, x = "Main.Groups", y = "pct.ct", color = "Braak") +
scale_y_continuous(labels=scales::percent)
ggsave("SEAAD_immuno_Distrib_boxplot_Braak_Main.Groups.pdf")
```

linear model to calculate changes in cell proportions

```
mtd<-data.table(SEAAD_immuno@meta.data,keep.rownames = "bc")
mtd[,Total:=.N,"donor_id"]
mtd[,Count:=.N,c("donor_id","Main.Groups")]
mtd[,Other:=Total-Count]
```

use GLM to estimate the proportions of the cell types across all the samples, treating the samples as replicates.

In this case a simple model can be made where Count vs Other only depends on the cell type identity

```
model0 <- glm(
  formula = cbind(Count, Other) ~ Main.Groups,
  family = binomial(link = 'logit'),
  data = mtd)

# obtain per-cell-type probability values with emmeans
library (emmeans)

emm0 <- emmeans(model0, specs = ~ Main.Groups)
emm0 %>%
  summary(infer = TRUE, type = 'response') %>%
  arrange(prob) -> cell_type_probs
cell_type_probs %>% head

# make predictors for cell type proportions that depend on predictors of interest
# as well as accounting for batch effects and making use of replicates.

mtd %>% filter(Braak %in% c('low', 'mid', "high")) -> df
formula = cbind(Count, Other) ~ Main.Groups * Braak + Main.Groups * APOE4.status +
  Main.Groups * Age.at.death + Main.Groups * sex
model1 <- glm(formula = formula, family = 'binomial', data = df)

# compare odds ratios of 'normal' vs 'dementia' for each 'Cluster' using emmeans

emm1 <- emmeans(model1, specs = revpairwise ~ Braak | Main.Groups)
emm1$contrasts %>%
  summary(infer = TRUE, type = 'response') %>%
  rbind() %>%
  as.data.frame() -> c_results

write.csv (c_results, "GLM_Braak_low_mid_high.csv")
```

```
# Same per Thal.phase score
mtd %>% filter(Thal.phase %in% c('Thal 0', 'Thal 1', "Thal 2", "Thal 3", "Thal 4", "Thal 5")) -> df
formula = cbind(Count, Other) ~ Main.Groups * Thal.phase + Main.Groups * APOE4.status +
  Main.Groups * Age.at.death + Main.Groups * sex
model1 <- glm(formula = formula, family = 'binomial', data = df)

# compare odds ratios of 'normal' vs 'dementia' for each 'Cluster' using emmeans
emm1 <- emmeans(model1, specs = revpairwise ~ Thal.phase | Main.Groups)
emm1$contrasts %>%
  summary(infer = TRUE, type = 'response') %>%
  rbind() %>%
  as.data.frame() -> c_results

write.csv (c_results, "GLM_Thal.csv")

# Same per APOE4.status
mtd %>% filter(APOE4.status %in% c('Y', 'N')) -> df
formula = cbind(Count, Other) ~ Main.Groups * APOE4.status + Main.Groups * disease +
  Main.Groups * Age.at.death + Main.Groups * sex
model1 <- glm(formula = formula, family = 'binomial', data = df)

# compare odds ratios of 'normal' vs 'dementia' for each 'Cluster' using emmeans
emm1 <- emmeans(model1, specs = revpairwise ~ APOE4.status | Main.Groups)
emm1$contrasts %>%
  summary(infer = TRUE, type = 'response') %>%
  rbind() %>%
  as.data.frame() -> c_results

# Same per disease
mtd %>% filter(disease %in% c('dementia', 'normal')) -> df
formula = cbind(Count, Other) ~ Main.Groups * disease + Main.Groups * APOE4.status +
```

```
  Main.Groups * Age.at.death + Main.Groups * sex
model1 <- glm(formula = formula, family = 'binomial', data = df)


# compare odds ratios of 'normal' vs 'dementia' for each 'Cluster' using emmeans
emm1 <- emmeans(model1, specs = revpairwise ~ disease | Main.Groups)
emm1$contrasts %>%
  summary(infer = TRUE, type = 'response') %>%
  rbind() %>%
  as.data.frame() -> c_results


#change of cells proportions using poisson
mtsl<-unique(mtd[Main.Groups%in%c("0","1","2","3","4","5",
"6","7","8","9","10","11","12")],by=c("donor_id","Main.Groups"))


glm.lin_hto<-stats::glm(n.cells.lin~n.cells+Main.Groups*Braak+APOE4.status+sex+
                    Age.at.death+Years.of.education+CERAD.score.score+ethnicity,family=poisson(),data =
mtsl)
summary(glm.lin_hto)


# By clustering, thresholds based on marker genes, or label transfer, cells in single-cell RNA-seq data
can be
#assigned cell type labels. One use of scRNA-seq data is to compare abundance of cell types between
experimental
# conditions or tissues. If a cell type is enriched in a disease condition it is an interesting avenue to
explore
# what causes this increase in abundance.
# Generalized linear models give a very simple yet powerful framework to study differences in cell
type abundance.
# Each cell type can be considered to be sampled from a population of cells in an experimental
sample.
# Using a binomial linear model one can analyse counts of repeated observations of binary choices.


## Identification of signaling pathways
```

```
# Cell chat
(https://htmlpreview.github.io/?https://github.com/sqjin/CellChat/blob/master/tutorial/CellChat-vignette.html)

## devtools::install_github("sqjin/CellChat")

library(CellChat)

library(patchwork)

options(stringsAsFactors = FALSE)


# Here we load a scRNA-seq data matrix and its associated cell meta data


data.input = SEAAD_immuno@assays$RNA@counts # normalized data matrix

meta = SEAAD_immuno@meta.data # a dataframe with rownames containing cell mata data

cell.use = rownames(meta)[meta$Braak == "low"] # extract the cell names from disease data


# Prepare input data for CelChat analysis

data.input = data.input[, cell.use]

meta = meta[cell.use, ]

# meta = data.frame(labels = meta$labels[cell.use], row.names = colnames(data.input)) # manually
create a dataframe consisting of the cell labels

unique(meta$Main.Groups) # check the cell labels

# Levels: Microglia DCs Tcells Proliferating Neutrophils Bcells


# Create a CellChat object

cellchat <- createCellChat(object = data.input, meta = meta, group.by = "Main.Groups")

#> [1] "Create a CellChat object from a data matrix"

# Set cell identities for the new CellChat object

# The cell groups used for CellChat analysis are  Glutamatergic Neurons GABAergic Neurons
Astrocytes Oligodendrocytes OPCs Microglia/PVM Endothelial cells VLMC SMC/Pericytes dying cells


# Add cell information into meta slot of the object (Optional)

cellchat <- addMeta(cellchat, meta = meta)

cellchat <- setIdent(cellchat, ident.use = "Main.Groups") # set "Main.Groups" as default cell identity
```

```r
levels(cellchat@idents) # show factor levels of the cell Main.Groups
groupSize <- as.numeric(table(cellchat@idents)) # number of cells in each cell group

# Set the ligand-receptor interaction database
CellChatDB <- CellChatDB.human # use CellChatDB.human if running on human data
showDatabaseCategory(CellChatDB)

# Show the structure of the database
dplyr::glimpse(CellChatDB$interaction)
#> Rows: 1,939
#> Columns: 11
#> $ interaction_name   <chr> "TGFB1_TGFBR1_TGFBR2", "TGFB2_TGFBR1_TGFBR2", "TGFB.
#> $ pathway_name       <chr> "TGFb", "TGFb", "TGFb", "TGFb", "TGFb", "TGFb", "TG.
#> $ ligand             <chr> "TGFB1", "TGFB2", "TGFB3", "TGFB1", "TGFB1", "TGFB2.
#> $ receptor           <chr> "TGFbR1_R2", "TGFbR1_R2", "TGFbR1_R2", "ACVR1B_TGFb.
#> $ agonist            <chr> "TGFb agonist", "TGFb agonist", "TGFb agonist", "TG.
#> $ antagonist         <chr> "TGFb antagonist", "TGFb antagonist", "TGFb antagon.
#> $ co_A_receptor      <chr> "", "", "", "", "", "", "", "", "", "", "", "", "",.
#> $ co_I_receptor      <chr> "TGFb inhibition receptor", "TGFb inhibition recept.
#> $ evidence           <chr> "KEGG: hsa04350", "KEGG: hsa04350", "KEGG: hsa04350.
#> $ annotation         <chr> "Secreted Signaling", "Secreted Signaling", "Secret.
#> $ interaction_name_2 <chr> "TGFB1 - (TGFBR1+TGFBR2)", "TGFB2 - (TGFBR1+TGFBR2).

# use a subset of CellChatDB for cell-cell communication analysis
# CellChatDB.use <- subsetDB(CellChatDB, search = "Secreted Signaling") # use Secreted Signaling
# use all CellChatDB for cell-cell communication analysis
CellChatDB.use <- CellChatDB # simply use the default CellChatDB

# set the used database in the object
cellchat@DB <- CellChatDB.use
```

```
# Preprocessing the expression data for cell-cell communication analysis

# subset the expression data of signaling genes for saving computation cost

cellchat <- subsetData(cellchat) # This step is necessary even if using the whole database

cellchat <- identifyOverExpressedGenes(cellchat)

cellchat <- identifyOverExpressedInteractions(cellchat)


# project gene expression data onto PPI (Optional: when running it, USER should set `raw.use = FALSE` in the function `computeCommunProb()` in order to use the projected data)

# cellchat <- projectData(cellchat, PPI.human)


# Compute the communication probability and infer cellular communication network

cellchat <- computeCommunProb(cellchat)


# Extract the inferred cellular communication network as a data frame

df.net <- subsetCommunication(cellchat) # returns a data frame consisting of all the inferred cell-cell communications at the level of ligands/receptors. Set slot.name = "netP" to access the the inferred communications at the level of signaling pathways


# df.net <- subsetCommunication(cellchat, sources.use = c(1,2), targets.use = c(4,5)) # gives the inferred cell-cell communications sending from cell groups 1 and 2 to cell groups 4 and 5.


# df.net <- subsetCommunication(cellchat, signaling = c("WNT", "TGFb")) # gives the inferred cell-cell communications mediated by signaling WNT and TGFb.


# Infer the cell-cell communication at a signaling pathway level

cellchat <- computeCommunProbPathway(cellchat)


cellchat <- netAnalysis_computeCentrality(cellchat)


# Calculate the aggregated cell-cell communication network

cellchat <- aggregateNet(cellchat)


# visualize the aggregated cell-cell communication network
```

```
groupSize <- as.numeric(table(cellchat@idents))

par(mfrow = c(1,2), xpd=TRUE)

netVisual_circle(cellchat@net$count, vertex.weight = groupSize, weight.scale = T, label.edge= F,
title.name = "Number of interactions")

netVisual_circle(cellchat@net$weight, vertex.weight = groupSize, weight.scale = T, label.edge= F,
title.name = "Interaction weights/strength")


# examine the signaling sent from each cell group

mat <- cellchat@net$weight

par(mfrow = c(3,4), xpd=TRUE)

for (i in 1:nrow(mat)) {

  mat2 <- matrix(0, nrow = nrow(mat), ncol = ncol(mat), dimnames = dimnames(mat))

  mat2[i, ] <- mat[i, ]

  netVisual_circle(mat2, vertex.weight = groupSize, weight.scale = T, edge.weight.max = max(mat),
title.name = rownames(mat)[i])

}


saveRDS(cellchat, file = "cellchat_SEAD_Braak_low.rds")

write.csv (df.net, "Cellchat_interactions_SEAD_Braak_low.csv")


## Identify and visualize outgoing communication pattern of secreting cells

library(NMF)

library(ggalluvial)


selectK(cellchat, pattern = "outgoing")


nPatterns = 6

cellchat <- identifyCommunicationPatterns(cellchat, pattern = "outgoing", k = nPatterns, height = 14)


# river plot

netAnalysis_river(cellchat, pattern = "outgoing")
```

```
# dot plot

netAnalysis_dot(cellchat, pattern = "outgoing")


## Repeat the same for Braak "mid"and "high" and then compare different conditions (https://htmlpreview.github.io/?https://github.com/sqjin/CellChat/blob/master/tutorial/Comparison_analysis_of_multiple_datasets.html)


#Load CellChat object of each dataset and then merge together

low <- read_csv("Cellchat_interactions_SEAD_Braak_low.csv")

high <- read_csv("Cellchat_interactions_SEAD_Braak_high.csv")


# Pairwise comparison

object.list <- list(high=high, low=low)

cellchat <- mergeCellChat(object.list, add.names = names(object.list))


# Compare the total number of interactions and interaction strength

gg1 <- compareInteractions(cellchat, show.legend = F, group = c(1,2))

gg2 <- compareInteractions(cellchat, show.legend = F, group = c(1,2), measure = "weight")

gg1 + gg2


# Differential number of interactions or interaction strength among different cell populations

# The differential number of interactions or interaction strength in the cell-cell communication network between two

# datasets can be visualized using circle plot, where red (or blue) colored edges represent increased (or decreased)

# signaling in the second dataset compared to the first one.

par(mfrow = c(2,4), xpd=TRUE)

netVisual_diffInteraction(cellchat, weight.scale = T)

netVisual_diffInteraction(cellchat, weight.scale = T, measure = "weight")


# same with heatmap only for LPS treated conditions

gg1 <- netVisual_heatmap(cellchat)
```

```
#> Do heatmap based on a merged object

gg2 <- netVisual_heatmap(cellchat, measure = "weight")

#> Do heatmap based on a merged object

gg1 + gg2


weight.max <- getMaxWeight(object.list, attribute = c("idents","count"))

par(mfrow = c(1,2), xpd=TRUE)

for (i in 1:length(object.list)) {

  netVisual_circle(object.list[[i]]@net$count, weight.scale = T, label.edge= F, edge.weight.max = weight.max[2], edge.width.max = 12, title.name = paste0("Number of interactions - ", names(object.list)[i]))

}


# Differential number of interactions or interaction strength among different cell types

group.cellType <- c(rep("Microglia"), rep("Tcells"), rep("DCs"), rep("Neutrophils"))

group.cellType <- factor(group.cellType, levels = c("Microglia", "Tcells", "DCs", "Neutrophils"))

object.list <- lapply(object.list, function(x) {mergeInteractions(x, group.cellType)})

cellchat <- mergeCellChat(object.list, add.names = names(object.list))

#> Merge the following slots: 'data.signaling','images','net', 'netP','meta', 'idents', 'var.features' , 'DB', and 'LR'.


weight.max <- getMaxWeight(object.list, slot.name = c("idents", "net", "net"), attribute = c("idents","count", "count.merged"))

par(mfrow = c(1,2), xpd=TRUE)

for (i in 1:length(object.list)) {

  netVisual_circle(object.list[[i]]@net$count.merged, weight.scale = T, label.edge= T, edge.weight.max = weight.max[3], edge.width.max = 12, title.name = paste0("Number of interactions - ", names(object.list)[i]))

}


# Comparing the outgoing and incoming interaction strength in 2D s


num.link <- sapply(object.list, function(x) {rowSums(x@net$count) + colSums(x@net$count)-diag(x@net$count)})
```

```
weight.MinMax <- c(min(num.link), max(num.link)) # control the dot size in the different datasets

gg <- list()

for (i in 1:length(object.list)) {

  gg[[i]] <- netAnalysis_signalingRole_scatter(object.list[[i]], title = names(object.list)[i], weight.MinMax = weight.MinMax)

}

#> Signaling role analysis on the aggregated cell-cell communication network from all signaling pathways

#> Signaling role analysis on the aggregated cell-cell communication network from all signaling pathways

patchwork::wrap_plots(plots = gg)


gg1 <- netAnalysis_signalingChanges_scatter(cellchat, idents.use = "Microglia")

#> Visualizing differential outgoing and incoming signaling changes from NL to LS

#> The following `from` values were not present in `x`: 0

#> The following `from` values were not present in `x`: 0, -1

gg2 <- netAnalysis_signalingChanges_scatter(cellchat, idents.use = "Tcells")

#> Visualizing differential outgoing and incoming signaling changes from NL to LS

#> The following `from` values were not present in `x`: 0, 2

#> The following `from` values were not present in `x`: 0, -1

patchwork::wrap_plots(plots = list(gg1,gg2))



##Identify the conserved and context-specific signaling pathways

cellchat <- computeNetSimilarityPairwise(cellchat, type = "functional")

#> Compute signaling network similarity for datasets 1 2

cellchat <- netEmbedding (cellchat, type = "functional")

#> Manifold learning of the signaling networks for datasets 1 2

cellchat <- netClustering(cellchat, type = "functional")

#> Classification learning of the signaling networks for datasets 1 2

# Visualization in 2D-space

netVisual_embeddingPairwise(cellchat, type = "functional", label.size = 3.5)
```

```
#> 2D visualization of signaling networks from datasets 1 2

#> Compare outgoing (or incoming) signaling associated with each cell population
library(ComplexHeatmap)
#> Loading required package: grid
#> ========================================
#> ComplexHeatmap version 2.10.0
#> Bioconductor page: http://bioconductor.org/packages/ComplexHeatmap/
#> Github page: https://github.com/jokergoo/ComplexHeatmap
#> Documentation: http://jokergoo.github.io/ComplexHeatmap-reference
#>
#> If you use it in published research, please cite:
#> Gu, Z. Complex heatmaps reveal patterns and correlations in multidimensional
#>   genomic data. Bioinformatics 2016.
#>
#> The new InteractiveComplexHeatmap package can directly export static
#> complex heatmaps into an interactive Shiny app with zero effort. Have a try!
#>
#> This message can be suppressed by:
#>   suppressPackageStartupMessages(library(ComplexHeatmap))
#> ========================================
i = 1
# combining all the identified signaling pathways from different datasets
pathway.union <- union(object.list[[i]]@netP$pathways, object.list[[i+1]]@netP$pathways)
ht1 = netAnalysis_signalingRole_heatmap(object.list[[i]], pattern = "outgoing", signaling = pathway.union, title = names(object.list)[i], width = 5, height = 6)
ht2 = netAnalysis_signalingRole_heatmap(object.list[[i+1]], pattern = "outgoing", signaling = pathway.union, title = names(object.list)[i+1], width = 5, height = 6)
draw(ht1 + ht2, ht_gap = unit(0.5, "cm"), height = nrow(ht1)*unit(2.5, "mm"))

ht1 = netAnalysis_signalingRole_heatmap(object.list[[i]], pattern = "incoming", signaling = pathway.union, title = names(object.list)[i], width = 5, height = 6, color.heatmap = "GnBu")
```

ht2 = netAnalysis_signalingRole_heatmap(object.list[[i+1]], pattern = "incoming", signaling = pathway.union, title = names(object.list)[i+1], width = 5, height = 6, color.heatmap = "GnBu")

draw(ht1 + ht2, ht_gap = unit(0.5, "cm"), height = nrow(ht1)*unit(2.5, "mm"))

ht1 = netAnalysis_signalingRole_heatmap(object.list[[i]], pattern = "all", signaling = pathway.union, title = names(object.list)[i], width = 5, height = 6, color.heatmap = "OrRd")

ht2 = netAnalysis_signalingRole_heatmap(object.list[[i+1]], pattern = "all", signaling = pathway.union, title = names(object.list)[i+1], width = 5, height = 6, color.heatmap = "OrRd")

draw(ht1 + ht2, ht_gap = unit(0.5, "cm"), height = nrow(ht1)*unit(2.5, "mm"))

Identify the upgulated and down-regulated signaling ligand-receptor pairs

netVisual_bubble(cellchat, sources.use = (1:2), targets.use = c(1:2), comparison = c(1, 2), angle.x = 45)

#> Comparing communications on a merged object

gg1 <- netVisual_bubble(cellchat, sources.use = (1:2), targets.use = c(1:2), comparison = c(1, 2), max.dataset = 1, title.name = "Increased signaling in AD", angle.x = 45, remove.isolate = T)

#> Comparing communications on a merged object

gg2 <- netVisual_bubble(cellchat, sources.use = (1:2), targets.use = c(1:2), comparison = c(1, 2), max.dataset = 2, title.name = "Decreased signaling in AD", angle.x = 45, remove.isolate = T)

#> Comparing communications on a merged object

gg1 + gg2

Identify dysfunctional signaling by using differential expression analysis

define a positive dataset, i.e., the dataset with positive fold change against the other dataset

pos.dataset = "high"

define a char name used for storing the results of differential expression analysis

features.name = pos.dataset

perform differential expression analysis

cellchat <- identifyOverExpressedGenes(cellchat, group.dataset = "datasets", pos.dataset = pos.dataset, features.name = features.name, only.pos = FALSE, thresh.pc = 0.1, thresh.fc = 0.1, thresh.p = 1)

#> Use the joint cell labels from the merged CellChat object

map the results of differential expression analysis onto the inferred cell-cell communications to easily manage/subset the ligand-receptor pairs of interest

net <- netMappingDEG(cellchat, features.name = features.name)

extract the ligand-receptor pairs with upregulated ligands in LS

net.up <- subsetCommunication(cellchat, net = net, datasets = "high",ligand.logFC = 0.2, receptor.logFC = NULL)

extract the ligand-receptor pairs with upregulated ligands and upregulated recetptors in NL, i.e.,downregulated in LS

net.down <- subsetCommunication(cellchat, net = net, datasets = "low",ligand.logFC = -0.1, receptor.logFC = -0.1)

gene.up <- extractGeneSubsetFromPair(net.up, cellchat)

gene.down <- extractGeneSubsetFromPair(net.down, cellchat)

pairLR.use.up = net.up[, "interaction_name", drop = F]

gg1 <- netVisual_bubble(cellchat, pairLR.use = pairLR.use.up, sources.use = (1:2), targets.use = c(1:2), comparison = c(1, 2), angle.x = 90, remove.isolate = T,title.name = paste0("Up-regulated signaling in ", names(object.list)[1]))

#> Comparing communications on a merged object

pairLR.use.down = net.down[, "interaction_name", drop = F]

gg2 <- netVisual_bubble(cellchat, pairLR.use = pairLR.use.down, sources.use = (1:2), targets.use = c(1:2), comparison = c(1, 2), angle.x = 90, remove.isolate = T,title.name = paste0("Down-regulated signaling in ", names(object.list)[1]))

#> Comparing communications on a merged object

gg1 + gg2

Chord diagram

par(mfrow = c(1,2), xpd=TRUE)

netVisual_chord_gene(object.list[[2]], sources.use = (1:2), targets.use = c(1:2), slot.name = 'net', net = net.up, lab.cex = 0.8, small.gap = 3.5, title.name = paste0("Up-regulated signaling in ", names(object.list)[1]))

netVisual_chord_gene(object.list[[1]], sources.use = (1:2), targets.use = c(1:2), slot.name = 'net', net = net.down, lab.cex = 0.8, small.gap = 3.5, title.name = paste0("Down-regulated signaling in ", names(object.list)[1]))

```
# visualize the enriched ligands in the first condition
computeEnrichmentScore(net.down, species = 'human')

# visualize the enriched ligands in the second condition
computeEnrichmentScore(net.up, species = 'human')

## Visually compare cell-cell communication using Hierarchy plot, Circle plot or Chord diagram
pathways.show <- c("GAS")
weight.max <- getMaxWeight(object.list, slot.name = c("netP"), attribute = pathways.show) # control the edge weights across different datasets
par(mfrow = c(1,2), xpd=TRUE)
for (i in 1:length(object.list)) {
  netVisual_aggregate(object.list[[i]], signaling = pathways.show, layout = "circle", edge.weight.max = weight.max[1], edge.width.max = 10, signaling.name = paste(pathways.show, names(object.list)[i]))
}

## Compare the signaling gene expression distribution between different datasets

cellchat@meta$datasets = factor(cellchat@meta$datasets, levels = c("high", "low")) # set factor level
plotGeneExpression(cellchat, signaling = "CXCL", split.by = "datasets", colors.ggplot = T)
#> The default behaviour of split.by has changed.
#> Separate violin plots are now plotted side-by-side.
#> To restore the old behaviour of a single split violin,
#> set split.plot = TRUE.
#>
#> This message will be shown once per session.
#> Scale for 'y' is already present. Adding another scale for 'y', which will
#> replace the existing scale.
#> Scale for 'y' is already present. Adding another scale for 'y', which will
#> replace the existing scale.
#> Scale for 'y' is already present. Adding another scale for 'y', which will
#> replace the existing scale.
```

```
saveRDS(cellchat, file = "cellchat_comparisonAnalysis_SEAAD_immuno_highvslow.rds")



# Study non-microglial clusters
Adaptative <- subset (SEAAD_immuno, Main.Groups %in% c("Microglia", "Proliferating"),
invert=TRUE)
Adaptative <- subset (Adaptative, Braak %in% c("low", "mid", "high"))
# 2132 cells

Adaptative <- RunPCA(Adaptative, features = VariableFeatures(object = Adaptative))
Adaptative <- RunUMAP(Adaptative, reduction = "pca", dims = 1:20)
Adaptative <- FindNeighbors(Adaptative, dims = 1:20)
Adaptative <- FindClusters(Adaptative, resolution = 1)

DimPlot(Adaptative, reduction = "umap", label=TRUE, split.by="Braak")

mtd<-data.table(Adaptative@meta.data,keep.rownames = "bc")
ggplot(mtd)+geom_bar(aes(x=Braak ,fill=seurat_clusters),position = "fill")

# find markers for every cluster compared to all remaining cells, report only the positive ones
Adaptative.markers <- FindAllMarkers(Adaptative, only.pos = TRUE, min.pct = 0.25, logfc.threshold =
0.25)
Adaptative.markers %>% group_by(cluster) %>% top_n(n = 2, wt = avg_log2FC)

# Top 10 genes
top5 <- Adaptative.markers %>% group_by(cluster) %>% top_n(n = 5, wt = avg_log2FC)
DoHeatmap(Adaptative, features = top5$gene, cells = 1:500) + NoLegend()

DotPlot (Adaptative, features=c("TOX","NKG7", "CCL5", "PDCD1","XCL", "LY6C2","ISG15", "FOLR4R",
"SLAMF6", "EGR1", "ITGAX", "KLRC2", "CCR7",
                    "SELL", "RELB", "FOXP3", "GZMA")) + coord_flip()
```

```
DotPlot(Adaptative, features=c("CD3D","CD3G", "CD4", "CD8A", "CD8B","FOXP3", "NKG7","GNLY", "CD19",
                        "JCHAIN",  "MRC1", "CD68", "CD14", "S100A8", "FCGR3A", "NR4A1"),
    cols = c("lightgrey", "darkred"), assay='RNA') + coord_flip()

# Based on these markers
# 0 - CD8+ T cells
# 1 to 4 - Macrophages
# 5 - CD8+ T cells
# 6 - NK cells
# 7 - Neutrophils
# 8 - Monocytes
# 9 - CD4+ T cells
# 10 - B cells

Adaptative$ordered <- Adaptative@meta.data$seurat_clusters
Adaptative@meta.data$ordered <- factor (Adaptative@meta.data$ordered, levels = c("0", "5", "9",
                            "6", "10", "1", "2", "3", "4", "7", "8"))
Idents(Adaptative)<- Adaptative@meta.data$ordered

DoHeatmap(Adaptative, features = top5$gene, cells = 1:500) + NoLegend()

# linear model to calculate changes in cell proportions

mtd<-data.table(Adaptative@meta.data,keep.rownames = "bc")
mtd[,Total:=.N,"donor_id"]
mtd[,Count:=.N,c("donor_id","ordered")]
mtd[,Other:=Total-Count]

#  use  GLM to estimate the proportions of the cell types across all the samples, treating the samples as replicates.
```

In this case a simple model can be made where Count vs Other only depends on the cell type identity

```
model0 <- glm(
  formula = cbind(Count, Other) ~ ordered,
  family = binomial(link = 'logit'),
  data = mtd
)
# obtain per-cell-type probability values with emmeans
library (emmeans)

emm0 <- emmeans(model0, specs = ~ ordered)
emm0 %>%
  summary(infer = TRUE, type = 'response') %>%
  arrange(prob) -> cell_type_probs
cell_type_probs %>% head

# make predictors for cell type proportions that depend on predictors of interest
# as well as accounting for batch effects and making use of replicates.

mtd %>% filter(Braak %in% c('low', 'mid', "high")) -> df
formula = cbind(Count, Other) ~ ordered * Braak + ordered * APOE4.status +
  ordered * Age.at.death + ordered * sex
model1 <- glm(formula = formula, family = 'binomial', data = df)

# compare odds ratios of 'normal' vs 'dementia' for each 'Cluster' using emmeans

emm1 <- emmeans(model1, specs = revpairwise ~ Braak | ordered)
emm1$contrasts %>%
  summary(infer = TRUE, type = 'response') %>%
  rbind() %>%
```

```
  as.data.frame() -> c_results

write.csv (c_results, "GLM_Braak_low_mid_high_Adaptative.csv")

# Cluster 5 in male and 0 in females
DimPlot(Adaptative, reduction = "umap", label=TRUE, split.by="sex")
DEGsclusters5vs0<-FindMarkers(Adaptative, ident.1 = 5, ident.2 = 0, test.use = "DESeq2")

VlnPlot (subset (Adaptative, seurat_clusters== c("0", "5")), features = c("CXCR6", "CCL5",
"NKG7","B2M","HLA-A",
                 "HLA-C", "IL7R", "XIST", "SYTL3"), assay="RNA")

# Study microglia clusters
Micro <- subset (SEAAD_immuno, Main.Groups =="Microglia")
# 36483 cells

Micro <- RunPCA(Micro, features = VariableFeatures(object = Micro))
Micro <- RunUMAP(Micro, reduction = "pca", dims = 1:20)
Micro <- FindNeighbors(Micro, dims = 1:20)
Micro <- FindClusters(Micro, resolution = 0.5)

DimPlot(Micro, reduction = "umap", label=TRUE, split.by="Braak")

mtd<-data.table(Micro@meta.data,keep.rownames = "bc")

# find markers for every cluster compared to all remaining cells, report only the positive ones
Micro.markers <- FindAllMarkers(Micro, only.pos = TRUE, min.pct = 0.25, logfc.threshold = 0.25)
Micro.markers %>% group_by(cluster) %>% top_n(n = 2, wt = avg_log2FC)
```

```
# Top 10 genes

top5 <- Micro.markers %>% group_by(cluster) %>% top_n(n = 5, wt = avg_log2FC)

DoHeatmap(Micro, features = top10$gene) + NoLegend()


# Cell-ID


library (CelliD)

fp<-function(...)file.path(...)


# Downsample Micro.rds


Micro_downsample <- subset (Micro, downsample=500)

# 10522 cells



#Run MCA

DefaultAssay(Micro_downsample)<-"SCT"

Micro_downsample <- RunMCA(Micro_downsample)




## Microglia signatures Keren-Shaul et al 2017

# Read compiled sheet in Keren-Shaul DAM gene list.xlsx


gene_set_list2<-split(Keren_Shaul_DAM_gene_list$gene,Keren_Shaul_DAM_gene_list$subtype)


DefaultAssay(Micro_downsample)<-"RNA"

DimPlot(Micro_downsample, reduction = "umap", label=T, split.by = "Braak")



# Assessing per-cell functional enrichment analyses
```

```
#need to be a list of gene set named by the pathway

#like this :

#KEGG <-
fgsea::gmtPathways("https://amp.pharm.mssm.edu/Enrichr/geneSetLibrary?mode=text&libraryName=KEGG_2019_Human")


HGT_Micro <- RunCellHGT(Micro_downsample, pathways = gene_set_list2, dims = 1:50)


# Create assay in seurat object

Micro_downsample@assays[["Micro_signature"]] <- CreateAssayObject(HGT_Micro)

DefaultAssay(Micro_downsample)<-"Micro_signature"


#valid signature

gs_of_interest2<-c("Homeostatic", "ARM", "DAM", "PAM")


FeaturePlot(Micro_downsample, gs_of_interest2,order=T, reduction = "umap",cols = c("lightgrey", "darkred"), max.cutoff = "q95",

        split.by= "Braak", pt.size=0.2)



#gene_set enrichment in microglia

#determine threshold to call a cell enriched for the signature

gs_int_dt<-
data.table(t(as.matrix(Micro_downsample@assays$Micro_signature@data[gs_of_interest2,])),keep.rownames = "cell")

gs_int_dt<-melt(gs_int_dt,id.vars = "cell",variable.name ="gene_set",value.name = "enrichment" )

for(gs in gs_of_interest2){

  print(gs)

  print(ggplot(gs_int_dt[gene_set==gs])+geom_density(aes(x=enrichment)))

  gs_int_dt[gene_set==gs,enrich.thr:=as.numeric(readline("threshold: "))]


}
```

```
# threshold: 1

gs_int_dt[,enriched:=enrichment>enrich.thr]
mtd<-data.table(Micro_downsample@meta.data,keep.rownames = "cell")
gs_int_dt_mtd<-merge(gs_int_dt,mtd[,.(cell,seurat_clusters)],by="cell")
gs_int_dt_mtd[,pct.enriched:=sum(enriched)/.N,by=.(seurat_clusters,gene_set)]

#Ploportion sig enriched
gs_int_dt_mtsl<-unique(gs_int_dt_mtd[enriched=="TRUE"],by=c("seurat_clusters","gene_set"))
ggplot(gs_int_dt_mtsl)+
  geom_col(aes(x=seurat_clusters,y=pct.enriched))+facet_grid("gene_set")+theme_minimal()+
scale_y_continuous(labels=scales::percent)

# Per disease
gs_int_dt[,enriched:=enrichment>enrich.thr]
mtd<-data.table(Micro_downsample@meta.data,keep.rownames = "cell")
gs_int_dt_mtd<-merge(gs_int_dt,mtd[,.(cell,seurat_clusters, disease)],by="cell")
gs_int_dt_mtd[,pct.enriched:=sum(enriched)/.N,by=.(seurat_clusters,gene_set, disease)]

#Ploportion sig enriched
gs_int_dt_mtsl<-unique(gs_int_dt_mtd[enriched=="TRUE"],by=c("seurat_clusters","gene_set",
"disease"))
ggplot(gs_int_dt_mtsl)+
  geom_col(aes(x=seurat_clusters,y=pct.enriched))+facet_grid(gene_set~disease)+theme_minimal()+
scale_y_continuous(labels=scales::percent)


# GLM for adaptative and innate response cells
# linear model to calculate changes in cell proportions
```

```
mtd<-data.table(Micro@meta.data,keep.rownames = "bc")

mtd[,Total:=.N,"donor_id"]

mtd[,Count:=.N,c("donor_id","seurat_clusters")]

mtd[,Other:=Total-Count]


# use GLM to estimate the proportions of the cell types across all the samples, treating the samples as replicates.

# In this case a simple model can be made where Count vs Other only depends on the cell type identity


model0 <- glm(

  formula = cbind(Count, Other) ~ seurat_clusters,

  family = binomial(link = 'logit'),

  data = mtd

)


# obtain per-cell-type probability values with emmeans

library (emmeans)


emm0 <- emmeans(model0, specs = ~ seurat_clusters)

emm0 %>%

  summary(infer = TRUE, type = 'response') %>%

  arrange(prob) -> cell_type_probs

cell_type_probs %>% head


# make predictors for cell type proportions that depend on predictors of interest

# as well as accounting for batch effects and making use of replicates.


mtd %>% filter(Braak %in% c('low', 'mid', "high")) -> df

formula = cbind(Count, Other) ~ seurat_clusters * Braak + seurat_clusters * APOE4.status +

  seurat_clusters * Age.at.death + seurat_clusters * sex

model1 <- glm(formula = formula, family = 'binomial', data = df)
```

```
# compare odds ratios of 'normal' vs 'dementia' for each 'Cluster' using emmeans

emm1 <- emmeans(model1, specs = revpairwise ~ Braak | seurat_clusters)
emm1$contrasts %>%
  summary(infer = TRUE, type = 'response') %>%
  rbind() %>%
  as.data.frame() -> c_results

write.csv (c_results, "Micro_GLM_Braak_low_mid_high.csv")

# Same per sex score
mtd %>% filter(sex %in% c('male', 'female')) -> df
formula = cbind(Count, Other) ~ seurat_clusters * sex + seurat_clusters * APOE4.status +
  seurat_clusters * Age.at.death + seurat_clusters * sex
model1 <- glm(formula = formula, family = 'binomial', data = df)

# compare odds ratios of 'normal' vs 'dementia' for each 'Cluster' using emmeans

emm1 <- emmeans(model1, specs = revpairwise ~ sex | seurat_clusters)
emm1$contrasts %>%
  summary(infer = TRUE, type = 'response') %>%
  rbind() %>%
  as.data.frame() -> c_results

write.csv (c_results, "Micro_GLM_sex.csv")

# Same per APOE4.status
mtd %>% filter(APOE4.status %in% c('Y', 'N')) -> df
formula = cbind(Count, Other) ~ seurat_clusters * APOE4.status + seurat_clusters * disease +
  seurat_clusters * Age.at.death + seurat_clusters * sex
```

```
model1 <- glm(formula = formula, family = 'binomial', data = df)

# compare odds ratios of 'normal' vs 'dementia' for each 'Cluster' using emmeans

emm1 <- emmeans(model1, specs = revpairwise ~ APOE4.status | seurat_clusters)
emm1$contrasts %>%
  summary(infer = TRUE, type = 'response') %>%
  rbind() %>%
  as.data.frame() -> c_results

write.csv (c_results, "Micro_GLM_APOE.csv")

# Same per disease
mtd %>% filter(disease %in% c('dementia', 'normal')) -> df
formula = cbind(Count, Other) ~ seurat_clusters * disease + seurat_clusters * APOE4.status +
  seurat_clusters * Age.at.death + seurat_clusters * sex
model1 <- glm(formula = formula, family = 'binomial', data = df)

# compare odds ratios of 'normal' vs 'dementia' for each 'Cluster' using emmeans

emm1 <- emmeans(model1, specs = revpairwise ~ disease | seurat_clusters)
emm1$contrasts %>%
  summary(infer = TRUE, type = 'response') %>%
  rbind() %>%
  as.data.frame() -> c_results

write.csv (c_results, "Micro_GLM_diagnosis.csv")
```

Supplementary Figure: Classification of immune cells based on cell type specific markers. (A) UMAP representation of the different cell clusters identified by unsupervised clustering. (B-C) Dot plots showing the expression of PTPRC (CD45), ITGAM (CD11B), TREM2, P2RY12, CD3D, CD3G, CD19, JCHAIN, MRC1, S100A8, FCGR3A, NR4A1 in clusters before (B) and after (C) cell cluster annotation.

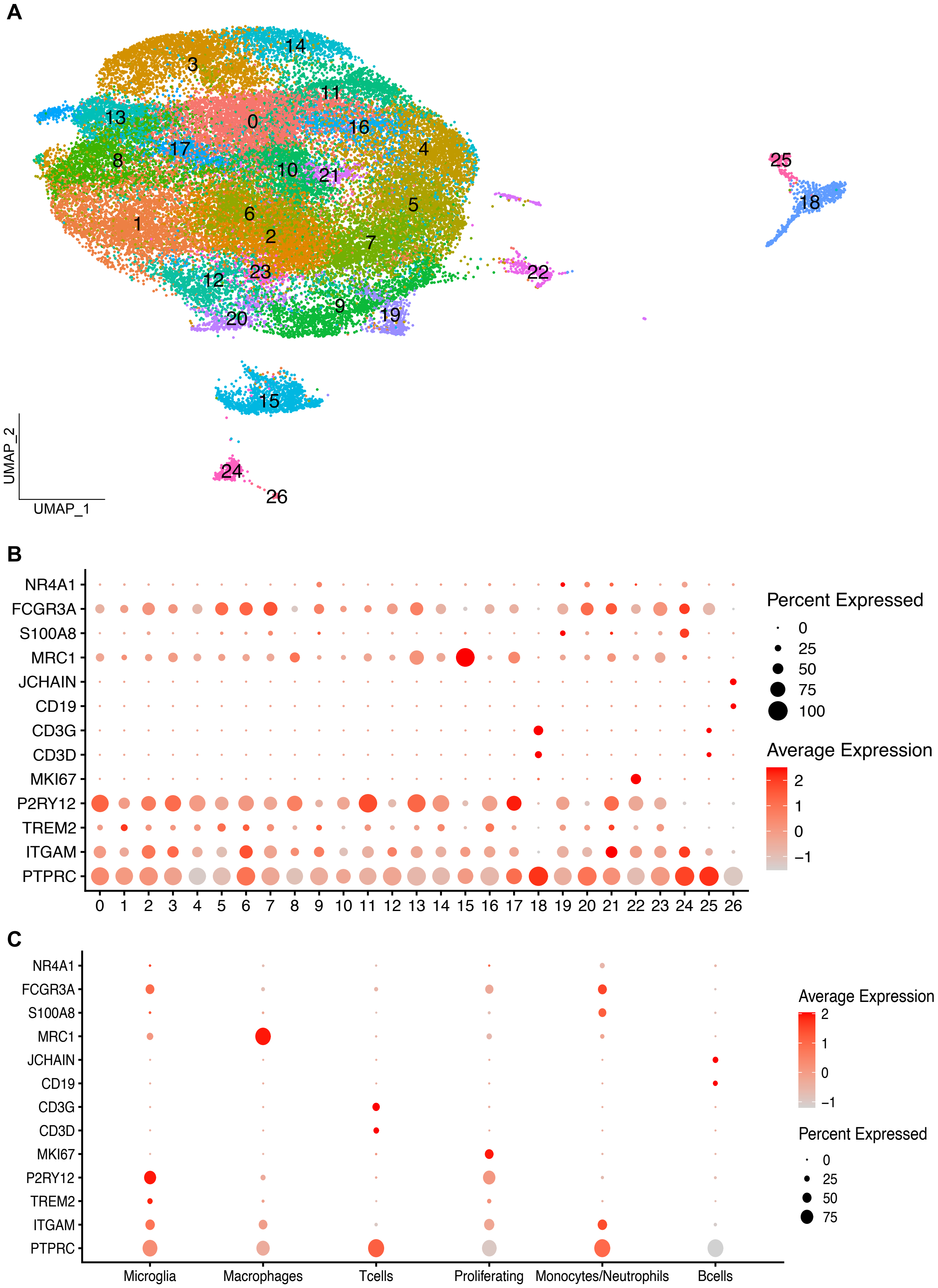
A
0
1
2
3
4
5
6
7
8
9
10
11
12
13
14
15
16
17
18
19
20
21
22
23
24
25
26
UMAP_2
UMAP_1
B
NR4A1
FCGR3A
S100A8
MRC1
JCHAIN
CD19
CD3G
CD3D
MKI67
P2RY12
TREM2
ITGAM
PTPRC
0 1 2 3 4 5 6 7 8 9 10 11 12 13 14 15 16 17 18 19 20 21 22 23 24 25 26
Percent Expressed
0
25
50
75
100
Average Expression
2
1
0
−1
C
NR4A1
FCGR3A
S100A8
MRC1
JCHAIN
CD19
CD3G
CD3D
MKI67
P2RY12
TREM2
ITGAM
PTPRC
Microglia
Macrophages
Tcells
Proliferating
Monocytes/Neutrophils
Bcells
Average Expression
2
1
0
−1
Percent Expressed
0
25
50
75
